# Supplementary material for: Self-contained Beta-with-Spikes approximation for inference under a Wright–Fisher model
Source: Genetics. 2023 May 25;225(2):iyad092. doi: 10.1093/genetics/iyad092 (PMC10550310; doi:10.1093/genetics/iyad092)
Supplement: iyad092_Supplementary_Data [file iyad092_supplementary_data.pdf]

OXFORD GENETICS

# Self-contained Beta-with-Spikes Approximation for Inference Under a Wright-Fisher Model

# Supplementary Methods

Juan Guerrero Montero[1,†,*] and Richard A. Blythe[1,†]

[1]SUPA, School of Physics and Astronomy, University of Edinburgh, Edinburgh, EH9 3FD, United Kingdom
[†]These authors contributed equally to this work.

*SUPA, School of Physics and Astronomy, University of Edinburgh, Edinburgh, EH9 3FD, United Kingdom. J.A.Guererro-Montero@sms.ed.ac.uk

## S1. Derivation of the estimation schemes

The estimation schemes each start with exact recursions for the mean and variance of the DAF at generation $t+k+1$. Given a transition probability $\mathrm{P}(x_{t+k}|x_t)$ representing the DAF after $k$ time steps with starting frequency $x_t$, the DAF at the next timestep can be found using the Chapman-Kolmogorov equation for the transition probabilities of the process:

$$\mathrm{P}(x_{t+k+1}|x_t) = \int \mathrm{Pr}_{\mathrm{WF}}(x_{t+k+1}|x_{t+k})\mathrm{P}(x_{t+k}|x_t)\mathrm{d}x_{t+k} \quad \text{(S.1)}$$

which arises from the law of total probability. In it, the one-step Wright-Fisher transition probability with population size $N$ is given by

$$\mathrm{Pr}_{\mathrm{WF}}(x'|x) = \binom{N}{Nx'} g\left(x\right)^{Nx'} \left(1-g\left(x\right)\right)^{N(1-x')} . \quad \text{(S.2)}$$

From this, the mean of the frequency at generation $t+k+1$ is given by

$$\begin{aligned} E_{k+1} &= \sum_{n=0}^{N} \frac{n}{N} \mathrm{P}\left(x_{t+k+1} = \frac{n}{N}|x_t\right) \\ &= \sum_{n=0}^{N} \frac{n}{N} \int \mathrm{Pr}_{\mathrm{WF}}\left(\frac{n}{N}|x_{t+k}\right) \mathrm{P}\left(x_{t+k}|x_t\right) \mathrm{d}x_{t+k} \\ &= \int \sum_{n=0}^{N} \frac{n}{N} \mathrm{Pr}_{\mathrm{WF}}\left(\frac{n}{N}|x_{t+k}\right) \mathrm{P}\left(x_{t+k}|x_t\right) \mathrm{d}x_{t+k} \\ &= \int g\left(x_{t+k}\right) \mathrm{P}\left(x_{t+k}|x_t\right) \mathrm{d}x_{t+k} \\ &= \mathbb{E}_{\mathrm{P}}\left[g(x_{t+k})\right] , \end{aligned} \quad \text{(S.3)}$$

where the third equality uses the analytical result for the mean of the Wright-Fisher transition probability, thus eliminating the explicit dependence of the final result on this probability function. In the last equality, $\mathbb{E}_{\mathrm{P}}$ represents the expectation under the transition probability P.

The variance, similarly, can be found as

$$\begin{aligned} V_{k+1} &= \sum_{n=0}^{N} \left(\frac{n}{N} - E_{k+1}\right)^2 \mathrm{P}\left(x_{t+k+1} = \frac{n}{N}|x_t\right) \\ &= \int \sum_{n=0}^{N} \left(\frac{n}{N}\right)^2 \mathrm{Pr}_{\mathrm{WF}}\left(\frac{n}{N}|x_{t+k}\right) \mathrm{P}(x_{t+k}|x_t)\mathrm{d}x_{t+k} - E_{k+1}^2 \\ &= \int \left[\left(1-\frac{1}{N}\right) g(x_{t+k})^2 + \frac{1}{N} g(x_{t+k})\right] \mathrm{P}(x_{t+k}|x_t)\mathrm{d}x_{t+k} - E_{k+1}^2 \\ &= \left(1-\frac{1}{N}\right)\left(\mathbb{E}_{\mathrm{P}}[g(x_{t+k})^2] - E_{k+1}^2\right) + \frac{1}{N}\left(\mathbb{E}_{\mathrm{P}}[g(x_{t+k})] - E_{k+1}^2\right) \\ &= \left(1-\frac{1}{N}\right) \mathrm{Var}_{\mathrm{P}}[g(x_{t+k})] + \frac{1}{N} \mathbb{E}_{\mathrm{P}}[g(x_{t+k})]\left(1 - \mathbb{E}_{\mathrm{P}}[g(x_{t+k})]\right) \end{aligned} \quad \text{(S.4)}$$

where the third equality uses the analytical form of the second moment of the Wright-Fisher transition probability, and the last equality uses equation S.3 together with the definition of the variance. In it, $\mathrm{Var}_{\mathrm{P}}$ represents the variance under the transition probability P.

The loss probability is given by

$$\begin{aligned} P_{0,k+1} =& \mathrm{P}\left(x_{t+k+1} = 0|x_t\right) \\ =& \int \mathrm{Pr}_{\mathrm{WF}}\left(0|x_{t+k}\right) \mathrm{P}(x_{t+k}|x_t)\mathrm{d}x_{t+k} \\ =& \int \left(1-g(x_{t+k})\right)^N \mathrm{P}(x_{t+k}|x_t)\mathrm{d}x_{t+k} \\ =& \mathbb{E}_{\mathrm{P}}\left[\left(1-g(x_{t+k})\right)^N\right] \end{aligned} \quad \text{(S.5)}$$

and equivalently for the fixation probability we have

$$\begin{aligned} P_{1,k+1} =& \mathrm{P}\left(x_{t+k+1} = 1|x_t\right) \\ =& \int \mathrm{Pr}_{\mathrm{WF}}\left(1|x_{t+k}\right) \mathrm{P}(x_{t+k}|x_t)\mathrm{d}x_{t+k} \\ =& \int g(x_{t+k})^N \mathrm{P}(x_{t+k}|x_t)\mathrm{d}x_{t+k} \\ =& \mathbb{E}_{\mathrm{P}}\left[g(x_{t+k})^N\right] \end{aligned} \quad \text{(S.6)}$$

### S1.1 Derivation of the truncated Taylor scheme

Equations S.3 to S.6 do not have closed analytical forms for arbitrary $g(x_{k+t})$, and their direct integration using a $k$-generation Wright-Fisher transition probability is computationally intractable in general. From equations S.3 to S.6, recursive relations for the moments, loss and fixation probabilities after $k+1$ generations can be obtained by Taylor expanding $g(x_{t+k})$ about $E_k$ up to second order, and dropping all moments of higher order than the variance.

For the mean (from equation S.3):

$$\begin{aligned}E_{k+1} \approx & g(E_k) + g'(E_k)\mathbb{E}_{\mathrm{P}}\left[(x_{t+k}-E_k)\right] \\ & + \frac{g''(E_k)}{2}\mathbb{E}_{\mathrm{P}}\left[(x_{t+k}-E_k)^2\right] \\ = & g(E_k) + \frac{g''(E_k)}{2}V_k \end{aligned} \tag{S.7}$$

where we have used the identities $\mathbb{E}_{\mathrm{P}}[x_{t+k}] = E_k$ and $\mathbb{E}_{\mathrm{P}}\left[(x_{t+k}-E_k)^2\right] = V_k$.

For the variance:

$$\begin{aligned}\mathrm{Var}_{\mathrm{P}}[g(x_{t+k})] = & \mathbb{E}_{\mathrm{P}}\left[g(x_{t+k})^2\right] - E_{k+1}^2 \\ \approx & g(E_k)^2 - E_{k+1}^2 + 2g'(E_k)g(E_k)\mathbb{E}_{\mathrm{P}}\left[(x_{t+k}-E_k)\right] \\ & + \left(g'(E_k)^2 + g''(E_k)g(E_k)\right)\mathbb{E}_{\mathrm{P}}\left[(x_{t+k}-E_k)^2\right] \\ = & -\left(\frac{g''(E_k)}{2}V_k\right)^2 - g''(E_k)g(E_k)V_k \\ & + \left(g'(E_k)^2 + g''(E_k)g(E_k)\right)V_k \\ = & -\left(\frac{g''(E_k)}{2}V_k\right)^2 + g'(E_k)^2V_k \\ \approx & g'(E_k)^2V_k \end{aligned} \tag{S.8}$$

where, in line with previous work (see supplementary material in Paris *et al.* (2019)), the last equality assumes $V_k^2$ to be of the same order as $\mathbb{E}_{\mathrm{P}}\left[(x_{t+k}-E_k)^4\right]$ and thus negligible. By introducing this result into equation S.4, we obtain

$$V_{k+1} \approx \frac{1}{N}E_{k+1}\left(1-E_{k+1}\right) + \left(1-\frac{1}{N}\right)V_k\, g'(E_k)^2 . \tag{S.9}$$

For the loss and fixation probabilities, Tataru *et al.* (2017) propose linearizing the fitness function as $g(x) = x$ and taking the transition probability $P(x_{t+k}|x_t)$ to be a Beta-with-Spikes:

$$\begin{aligned}\mathrm{Pr}_{\mathrm{BwS}}(x_{t+k}|x_t) = & P_{0,k}\delta(x_{t+k}) + P_{1,k}\delta(1-x_{t+k}) \\ & + (1-P_{1,k}-P_{0,k})\frac{x_{t+k}^{\alpha_k-1}(1-x_{t+k})^{\beta_k-1}}{\mathrm{B}(\alpha_k,\beta_k)} ,\end{aligned} \tag{S.10}$$

With that, starting from equation S.5:

$$\begin{aligned}P_{0,k+1} \approx & \mathbb{E}_{\mathrm{BwS}}\left[(1-x_{t+k})^N\right] \\ = & P_{0,k} + (1-P_{0,k}-P_{1,k})\int \frac{x^{\alpha_k+1}(1-x)^{\beta_k+N+1}\mathrm{d}x}{B(\alpha_k,\beta_k)} \\ = & P_{0,k} + (1-P_{0,k}-P_{1,k})\frac{B(\alpha_k,\beta_k+N)}{B(\alpha_k,\beta_k)} \end{aligned} \tag{S.11}$$

and similarly for the fixation probability (equation S.6):

$$P_{1,k+1} \approx P_{1,k} + (1-P_{0,k}-P_{1,k})\frac{B(\alpha_k+N,\beta_k)}{B(\alpha_k,\beta_k)} . \tag{S.12}$$

### S1.2 Derivation of the self-contained scheme

We take the intermediate distribution at time $t+k$ to be given by

$$\mathrm{Pr}_{\mathrm{int}}(x_{t+k+1}|x_t) = \int \mathrm{Pr}_{\mathrm{WF}}(x_{t+k+1}|x_{t+k})\mathrm{Pr}_{\mathrm{BwS}}(x_{t+k}|x_t)\mathrm{d}x_{t+k} . \tag{S.13}$$

With this, the integrals in equations S.3 to S.6 are computationally tractable without having to resort to approximations of the fitness function.

From equation S.3, the mean of the intermediate distribution with population size $N$ is given by

$$\begin{aligned}E_{k+1} &= \mathbb{E}_{\mathrm{BwS}}\left[g(x_k)\right] \\ &= P_{1,k} + (1-P_{0,k}-P_{1,k})\frac{\int g(x)x^{\alpha_k-1}(1-x)^{\beta_k-1}}{\mathrm{B}(\alpha_k,\beta_k)} ,\end{aligned} \tag{S.14}$$

where the last equality uses equation S.10 together with the assumptions $g(0) = 0$ and $g(1) = 1$.

The variance, similarly, can be found as

$$\begin{aligned}V_{k+1} = & \left(1-\frac{1}{N}\right)\left(\mathbb{E}_{\mathrm{BwS}}[g(x_{t+k})^2] - \mathbb{E}_{\mathrm{BwS}}[g(x_{t+k})]^2\right) \\ & + \frac{1}{N}\mathbb{E}_{\mathrm{BwS}}[g(x_{t+k})]\left(1-\mathbb{E}_{\mathrm{BwS}}[g(x_{t+k})]\right) \\ = & \left(1-\frac{1}{N}\right)\left[P_{1,k} + (1-P_{0,k}-P_{1,k})\frac{\int g(x)^2x^{\alpha_k-1}(1-x)^{\beta_k-1}}{\mathrm{B}(\alpha_k,\beta_k)}\right] \\ & + \frac{1}{N}E_{k+1} - E_{k+1}^2 ,\end{aligned} \tag{S.15}$$

where equations S.4, S.10 and S.14 have been used.

The loss probability (equation S.5) is given by

$$\begin{aligned}P_{0,k+1} = & \mathbb{E}_{\mathrm{BwS}}\left[(1-g(x_{t+k}))^N\right] \\ = & P_{0,k} + (1-P_{0,k}-P_{1,k})\frac{\int (1-g(x))^N x^{\alpha_k-1}(1-x)^{\beta_k-1}}{\mathrm{B}(\alpha_k,\beta_k)}\end{aligned} \tag{S.16}$$

and equivalently for the fixation probability (equation S.6) we have

$$\begin{aligned}P_{1,k+1} = & \mathbb{E}_{\mathrm{P}}\left[g(x_{t+k})^N\right] \\ = & P_{1,k} + (1-P_{0,k}-P_{1,k})\frac{\int g(x)^N x^{\alpha_k-1}(1-x)^{\beta_k-1}}{\mathrm{B}(\alpha_k,\beta_k)}\end{aligned} \tag{S.17}$$

These parameters may be used now to generate the parameters $\alpha_{k+1}$ and $\beta_{k+1}$ of the Beta-with-Spikes transition probability after $k+1$ generations.

## S2. Numerical integration techniques

To implement the self-contained Beta-with-Spikes approximation, we are required to evaluate integrals of the general form

$$I = \int_0^1 \frac{x^{\alpha-1}(1-x)^{\beta-1}}{(1+(e^s-1)x)^{\gamma}}\mathrm{d}x . \tag{S.18}$$

Specialized libraries may be able to deal with these. For people wishing to implement them in programming languages that do not have these libraries available, some care is needed in their computation. We set out these details below.

Depending on the values of $\alpha$ and $\beta$, the integrand may diverge at either endpoint, or be sharply peaked at some point $0 < x < 1$. Special handling is needed around these points.

More precisely, when $\alpha < 1$, the integrand diverges as $x \to 0$. In this situation, we split the range of integration at $x = \Delta x$, and expand the integral over $0 < x < \Delta x$ in powers of $\Delta x$ to second order. We find

$$\int_0^{\Delta x} \frac{x^{\alpha-1}(1-x)^{\beta-1}}{(1+(e^s-1)x)^\gamma}\mathrm{d}x \approx \frac{1}{\alpha}\Delta x^\alpha - \frac{\gamma S + \beta - 1}{\alpha+1}\Delta x^{\alpha+1} + \frac{(\beta-1)\gamma S + \frac{1}{2}(\beta-1)(\beta-2) + \frac{1}{2}S^2\gamma(\gamma+1)}{\alpha+2}\Delta x^{\alpha+2} \quad \text{(S.19)}$$

where $S = e^s - 1$. Similarly, when $\beta < 1$, there is a divergence as $x \to 1$ which can be handled by splitting the integral at $x = 1 - \Delta x$. The corresponding expansion is

$$\int_{1-\Delta x}^{1} \frac{x^{\alpha-1}(1-x)^{\beta-1}}{(1+(e^s-1)x)^\gamma}\mathrm{d}x \approx \frac{1}{\beta e^{-\gamma s}}\Delta x^\beta - \frac{\gamma\tilde{S} + \alpha - 1}{(\beta+1)e^{-\gamma s}}\Delta x^{\beta+1} + \frac{(\alpha-1)\gamma\tilde{S} + \frac{1}{2}(\alpha-1)(\alpha-2) + \frac{1}{2}\tilde{S}^2\gamma(\gamma+1)}{(\beta+2)e^{-\gamma s}}\Delta x^{\beta+2} \quad \text{(S.20)}$$

where $\tilde{S} = 1 - e^{-s}$. The integral is split at both boundaries when $\alpha < 1$ and $\beta < 1$.

The value of $\Delta x$ is obtained numerically as the distance from the boundary at which the integrand reaches a fixed, high value (1000 in our implementation) to ensure $\Delta x$ is small enough to make the previous Taylor expansions accurate, as this ensures $\Delta x \ll \frac{1}{1000}$. The rest of the integral is computed using trapezoid rule with adaptive quadrature (Press *et al.* 2007).

When $\alpha > 1, \beta > 1$ and $s \neq 0$, the derivative of the integrand has four roots, located at $x = 0$, $x = 1$ and

$$x_\pm = \frac{A \pm \sqrt{A^2 + 4(\alpha-1)B}}{2B} \quad \text{(S.21)}$$

where

$$A = (\alpha-1)e^s - (\beta-1) - (e^s-1)\gamma \quad \text{(S.22)}$$
$$B = (e^s-1)(\alpha-1+\beta-1-\gamma) \quad \text{(S.23)}$$

Since the integrand is always 0 at $x = 0$ and $x = 1$ and is positive, continuous and differentiable in between these two values, it must have and odd number of maxima in the $(0,1)$ interval. However, since there are only two stationary points in this interval, the integrand has only a single maximum, located at either $x_+$ or $x_-$.

To increase accuracy when this maximum is strongly peaked, the integral is split into two at the maximum, each half being computed using trapezoid rule with adaptive quadrature. This assures that the maximum is not missed by the adaptive rule in scenarios where it is narrower than the initial step, and slightly increases the speed of convergence of the integration method.

Finally, when $\alpha > 1, \beta > 1$ and $s = 0$, there is a maximum at

$$x_0 = \frac{\alpha-1}{\alpha+\beta-2}\,. \quad \text{(S.24)}$$

This can be handled in the same way as in the $s \neq 0$ case.

## S3. Sampling error equalisation

When dealing with time-series data, estimates of an allele's (or cultural variant's) frequency may derive from samples of different sizes at different times, and therefore be subject to greater or lesser degrees of sampling error. To disentangle this from fluctuations in the underlying frequencies themselves (arising, for example, from genetic drift), it is helpful to equalize the amplitude of the sampling error across the time series. Then, any changes in the amplitude of the resulting fluctuations over time can be ascribed to the process that generates the underlying frequencies, as changes in how samples are constructed have already been accounted for.

In the main text, we describe a resampling procedure that effects this equalization. Given a sample of size $n$ at some time $t$, within which a fraction $x$ of items are of one particular variant (i.e., a specific allele or word form), we construct a binomial sample of size $m$ and success probability $x$ in such a way that the variance of the corresponding variant frequency $y$ is consistent with being derived from a binomial sample of fixed size $m_0$. The key point to note here is that the original sampling process already contributes some variance to $y$. Therefore, $m$ will depend on the original sample size so that the additional variance arising from resampling gives the desired overall variance.

To determine the appropriate sample size $m$, we consider the first two moments of the random variable $y$. Given some value of $x$ via the original sampling process, the binomial resampling process implies that

$$\mathbb{E}(y|x) = x \quad \text{(S.25)}$$
$$\mathbb{E}(y^2|x) = \left(1 - \frac{1}{m}\right)x^2 + \frac{1}{m}x\,. \quad \text{(S.26)}$$

We now average over all possible realizations of the original sampling process to determine the first two moments of the resampled frequency $y$, finding

$$\mathbb{E}(y) = \mathbb{E}(x) \quad \text{(S.27)}$$
$$\mathbb{E}(y^2) = \left(1 - \frac{1}{m}\right)\mathbb{E}(x^2) + \frac{1}{m}\mathbb{E}(x)\,. \quad \text{(S.28)}$$

Although the true variant frequency $p$ is unknown, we have that

$$\mathbb{E}(x) = p \quad \text{(S.29)}$$
$$\mathbb{E}(x^2) = \left(1 - \frac{1}{n}\right)p^2 + \frac{1}{n}p \quad \text{(S.30)}$$

where $n$ is the original sample size. Substituting these expressions into (S.27) and (S.28), we find that

$$\mathrm{Var}(y) = \mathbb{E}(y^2) - [\mathbb{E}(y)]^2 \quad \text{(S.31)}$$
$$= \left[1 - \left(1 - \frac{1}{n}\right)\left(1 - \frac{1}{m}\right)\right]p(1-p)\,. \quad \text{(S.32)}$$

This is the variance that would be obtained if the original sampling process involved a sample of size

$$\frac{1}{m_0} = 1 - \left(1 - \frac{1}{n}\right)\left(1 - \frac{1}{m}\right) \quad \text{(S.33)}$$

where $m_0$ is the fixed effective sample size introduced above. We note that this result can also be obtained by applying the law of total variance to the pair of random variables $x$ and $y$, where $x$ is drawn from $\mathrm{Bin}(n,p)$ and $y$ from $\mathrm{Bin}(m,x)$.

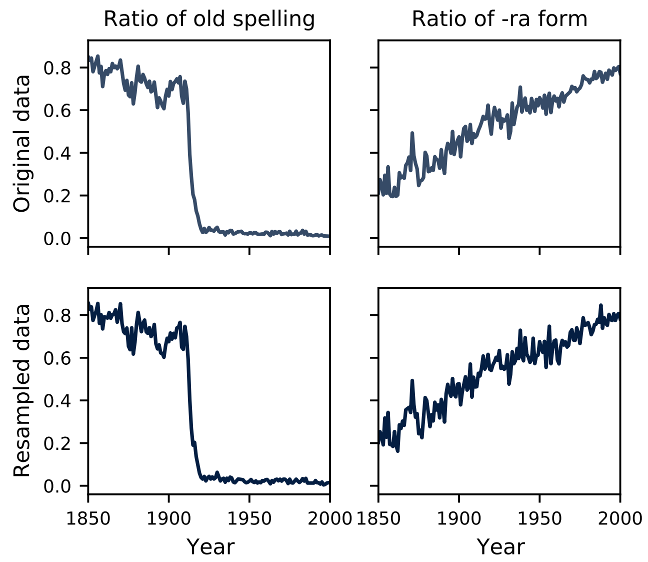


**Figure S1** Comparison of the Google Books Spanish time series before (upper panels) and after (lower panels) sampling error equalization. Left panels relate to the ratio of old spelling of single-letter words (*a*, *e*, *o*, *u*) and the right panels to the ratio of usage of the *-ra-* form of the past subjunctive, as opposed to the completely equivalent *-se-* form. The effect of the sampling error equalization is particularly evident in the case of the *-ra-* form.

Rearranging, we find that the resampled population size $m$ should be

$$m = \frac{1 - \frac{1}{n}}{\frac{1}{m_0} - \frac{1}{n}} \approx \frac{m_0}{1 - \frac{m_0}{n}} , \tag{S.34}$$

in which the approximation holds when the original sample size $n \gg 1$, which is typically the case. This latter approximate formula is the one that is quoted in the main text.

The effects of resampling on the Google Books Spanish data can be observed in Figure S1. The effects of noise equalization are particularly noticeable in the last 50 years of the ratio of the -ra form data set (right panels). Without applying the equalization (upper right panel), a spurious change in effective population size is detected. After applying the equalization, we find that the fluctuations maintain a similar amplitude throughout the time series, and no significant change in effective population size is found (as reported in the main text).

## Literature cited

Paris C, Servin B, Boitard S. 2019. Inference of selection from genetic time series using various parametric approximations to the wright-fisher model. G3 Genes | Genomes | Genetics. 9:4073–4086.

Press WH, Teukolsky SA, Vetterling WT, Flannery BP. 2007. *Numerical Recipes: The Art of Scientific Computing*. Cambridge University Press. Cambridge. third edition.

Tataru P, Simonsen M, Bataillon T, Hobolth A. 2017. Statistical inference in the wright–fisher model using allele frequency data. Systematic Biology. 66:e30–e46.
